# Compulsive Internet Use Scale for assessment of self-reported problematic internet use in primary school-aged children

**DOI:** 10.3389/fpsyt.2023.1173585

**Published:** 2023-06-30

**Authors:** Roma Jusienė, Vilmantė Pakalniškienė, Jennifer Chun-Li Wu, Sandra B. Sebre

**Affiliations:** ^1^Institute of Psychology, Vilnius University, Vilnius, Lithuania; ^2^Department of Early Childhood and Family Education, National Taipei University of Education, Taipei, Taiwan; ^3^Department of Psychology, University of Latvia, Riga, Latvia

**Keywords:** CIUS, internet use, children, multiple informants, psychometric validation

## Abstract

**Introduction:**

The tremendous growth of internet use during past few decades has been primarily led by young people. Despite a plenitude of studies reporting the pros and cons of excessive internet use by adolescents, the internet use of primary school-aged children is under-researched. First, there is lack of reliable and valid cultural invariant self-report instruments for children younger than 11-years-old. Secondly, there is no consensus on whether primary school-aged children can reliably report on their internet use. This study aimed to examine the psychometric properties of the Compulsive Internet Use Scale (CIUS) as reported by primary school-aged children in three different countries/regions.

**Methods:**

Paper-pencil format CIUS questionnaires were completed by a total of 691 children aged 8 to 10 years old, 236 of them Latvian, 207 Lithuanian, and 248 Taiwanese, as well as by one of their parents, at two-time points, separated by a one-year interval. The parents also reported on the child’s emotional and behavioral difficulties.

**Methods:**

Confirmatory factor analysis indicated that for the child self-report, a 10-item CIUS showed the best fit and good psychometric properties: solid structural validity; very good internal consistency; appropriate stability and predictive validity after 1 year; as well as sound sensitivity and specificity when compared to the 14-item CIUS parent-report form. Child self-report CIUS ratings correlated with time online reported by the child and parent and with emotional and behavioral problems reported by the parent.

**Discussion:**

This study indicates that children as young as 8–10 years old can reliably and consistently provide valuable information on their problematic use of the internet.

## Introduction

There is no doubt that internet has become a necessary attribute of everyday life, the main tool for social network building, communication, education, services, entertainment activities, etc. The tremendous growth of internet use during the past few decades has been primarily led by young people, particularly adolescents and emerging adults, as noted in many parts of the world (1–4). It is known that already primary school-aged children are proficient internet users, with a significant portion of them using the internet unattended by parents (5–7). The increasing amount of time that people spend online raises the question as to whether, for some individuals, internet use has become beyond their control (8), problematic (9) or “maladaptive” (10). This question seems particularly relevant regarding children, who are still developing their self-control and might be more vulnerable to environmental effects. Thus, there is a need to have the reliable and valid instruments to assess the problematic use of the internet in youngsters.

For more than two decades, studies on internet-related addiction have been on the rise. Various terms have been used to describe this phenomenon in clinical and non-clinical samples: pathological Internet use, problematic Internet use, excessive Internet use, or compulsive Internet use (8, 9, 11–14). Recently the term “Problematic Use of the Internet” (PUI) has been often used in scientific literature (4), although there is no standardized definition of the PUI among researchers (9). To summarize several comprehensive debates and reviews (4, 9, 10, 15, 16), PUI refers to the excessive and disproportionate engagement in various online activities (such as online gaming, shopping, social networking, etc.), characterized by a loss of control and the maintenance of the behavior despite negative consequences. While PUI has not been recognized as an official disorder and lacks agreed-upon diagnostic criteria (17), the addictive and compulsive components of PUI are continuously highlighted (9, 18).

There is no clear consensus by researchers on the predictors of internet-related addictive behavior, yet at least two correlates have been consistently identified. First, extensive internet use has been linked to young people’s mental health problems, e.g., anxiety, depression, and hyperactivity (1, 8, 17, 19–23), although there are contradictory conclusions as to whether excessive internet use is a consequence or antecedent of the mental health issues (1, 24). An extensive study, including data from 25 European countries, indicated that children’s excessive internet use might be a symptom of broader behavioral problems (25). Second, PUI has been significantly related to time spent online (14, 20, 22). This is not a criterion of internet addiction *per se*; however, a high level of internet use intensity and frequency may facilitate internet-related addictive behavior (8, 15, 26, 27). In the present study we highlight the need to identify problematic internet use in preadolescent children, and we aim to focus on validating the Compulsive Internet Use Scale (CIUS) (14) as completed by primary school-aged children. We propose that the validation of an instrument measuring the child’s problematic internet-use behavior should include an analysis of associations with the duration of time spent online, as well as associations with the child’s emotional and behavioral problems.

There have been many different instruments used to measure PUI and related constructs (9, 17, 28). The Internet Addiction Test (IAT) developed by Young is the most known and has the longest history (4). It has been widely used in research and clinical practice, but mostly with teenagers and adults. Several other instruments to screen for PUI (e.g., Generalized Problematic Internet Use Scale-2 developed by Caplan, the Problematic Internet Use Questionnaire developed by Demetrovics et al. or shorter and more recent PIUQ-9 by Laconi et al.) have been adapted and validated also with older adolescents and adults (9, 17, 29). One of the most widely used instruments to assess PUI is the relatively brief and concise 14-item CIUS developed by Meerkerk (14, 30) which was primarily designed to capture the thoughts and behaviors related to one’s inability to control one’s online activity. The CIUS is characterized by five main features, also prominent in recent definitions of PUI: (1) loss of control, e.g., continuation of internet use despite the intention to stop; (2) preoccupation, e.g., internet use dominating the user’s cognitions and behaviors; (3) withdrawal symptoms, e.g., the experience of unpleasant emotions when internet use is not possible; (4) a means of coping, e.g., using the internet to escape from negative feelings; and (5) conflict, e.g., internet use resulting in conflict with others or within oneself (14, 30). The composite (sum) score of the CIUS items represents the individual’s risky, problematic or compulsive internet use. It has been noted that the CIUS has various advantages over other instruments for screening internet-related addictive behaviors, such as suitability for research and clinical applications, the economization of time due to the limited number of questionnaire items, and the ease of use for online studies (26, 28, 31). In addition, it is among the most widely used instruments in longitudinal PUI research (9, 24).

During the past decade of intensive internet-related research, the CIUS has proven to be a well-validated self-report tool with good psychometric properties in terms of reliability and validity, with most studies supporting a one-factor solution when used with adolescents and adults across different cultures and languages (4, 27, 28, 31–37). Moreover, the CIUS has been shown to be relatively gender and age invariant (3, 12, 14, 35). Various shortened versions of the CIUS have been developed during the past several years (1, 12, 20, 38). Lopez-Fernandez et al. compared four versions (CIUS-14, CIUS-9, CIUS-7, and CIUS-5) across eight languages in 15 countries and demonstrated the validity of all four versions in all eight languages (26). In addition, the short forms of the CIUS were also confirmed as reliable and valid with a sample of Lithuanian medical students (39). As far as we know, up until this present study the youngest respondents reporting their internet-related compulsive behavior with the CIUS have been children in late childhood or early adolescence, either 11 (20, 27) or 12 years of age (33, 36). In these previous studies the full 14-item version or the shortened 10-item version of the CIUS (36) has been used and validated.

Dhir et al. (33, 34) have noted that internet use researchers should further examine the CIUS items to determine if any adaptations to this instrument are needed to address specific internet user groups. Beyond doubt, among the most important groups of internet users are young school-aged children. First, many primary school-aged children in many countries worldwide have their own smartphones or other devices with which they can gain access to the internet (40). Second, a considerable number of school-aged children use the internet unsupervised by parents (7). Despite this, the problematic internet use in children still lacks sufficient attention of researchers, possibly because there is no consensus on who should be reporting the internet use duration and the compulsivity of internet use of school-aged children younger than 11 years old. Is it possible for primary school-aged children to reliably report on their internet use?

Previous research on related topics has shown that children 11 years old and older are considered reliable informants on their mental health difficulties, e.g., emotional and behavioral problems, loneliness, etc. (41–43). Yet, several studies have indicated sufficient reliability and validity of emotional and behavioral problem self-report by younger children, e.g., aged 7 to 10 years (44, 45). Riley (46) has claimed that starting from 6 years of age, and with greater confidence from 8 years of age, children can provide valuable and reliable information on their own health issues. Similarly, Varni et al. (47) have reported that children as young as 5 years old can reliably and validly report on their health-related quality of life when provided with age-appropriate instruments. In addition, Measelle et al. (48) demonstrated that children aged 6 to 7 years could reliably report on their personality traits. Although often parental reports are used when young children’s health and behavioral issues are being addressed, it has been demonstrated that the information from parental proxy reports is not equivalent to that reported by the child (47). Especially since the construct of PUI involves not only observable behaviors but also mental thought processes, validating children’s subjective perception and appraisal seems essential. Thus, we propose that for research and clinical assessment purposes, it is important to have reliable and valid tools for screening children for risk of internet-related addiction, e.g., highly problematic use of the internet. The CIUS forms allowing one to obtain comprehensive multi-informant reports could be particularly valuable, especially when surveying young internet consumers.

In this study we aimed to provide a comprehensive analysis of the psychometric characteristics of the child self-report and parent-report CIUS ratings of primary school-aged children’s problematic internet use. Whereas the main focus of the present study is on the child self-report CIUS as a reliable and valid measure of their problematic internet use, we aim to show that the child self-report CIUS could have good construct stability in time (over 1 year), and could be well predicted by parent-report CIUS. We also hypothesize that child reported PUI should be significantly related to children’s emotional and behavioral problems reported by parents, as well as by time spent online, as reported by parents and the children themselves.

Since the data for this study were obtained using the same study protocol in three different countries/regions (Latvia, Lithuania, and Taiwan), we were able to examine the reliability and validity of the CIUS as completed by parents and children 8–10 years old in three different languages and cultures. All three are high-income regions, with comparable and high internet access (40). However, some differences in the structure of the school systems at the time of data gathering were notable, for example, children in Latvia and Lithuania typically began first grade at age 7 and in Taiwan—at age 6. Information and technology (IT) literacy as a core competency was included in the curriculum as obligatory from 2nd grade in Taiwan but only from 5th to 7th grade in Latvia and Lithuania. Compared to Latvia and Lithuania, Taiwan had a more progressive policy and practice agenda for preventing children’s problematic internet use (40). Previously published results of our joint international study conducted have shown similar risks of problematic internet use, yet considerable differences in the amount of time spent online and mean rates of CIUS (22, 40). Thus, investigating the psychometric properties of the CIUS in these three different languages/cultures is valuable for distal context-related and cross-cultural comparisons. Most cross-cultural validation research of the CIUS has been among European countries, whereby this study involves cross-continental comparison.

## Materials and methods

### Participants

The data for this study comes from a cross-cultural collaborative one-year longitudinal study conducted during the time period from 2018 to 2019 [for more information (22, 40)]. Respondents included 691 child–parent dyads (*N* = 236 from Latvia; *N* = 207 from Lithuania; and *N* = 248 from Taiwan) who completed questionnaires at two time points, separated by a one-year interval. At Time 1 (autumn of 2018; T1) the children were primarily 8 to 10 years old (mean age 8.78, *SD* = 0.72; 51.8% girls). The children attended typical public schools from a major city in each country, excluding schools with a specialized focus. Two-thirds of the participating parents had university education (see Table 1). A comparison of samples between countries showed that the male–female ratio in each sample was not significantly different (*χ^2^* = 2.34, *p* = 0.310), but there were differences according to the children’s and parents’ age (*F* = 141.13, *p* < 0.001; *F* = 109.18, *p* < 0.001). Parental age differed between all three samples, and the children’s average age in the Taiwan sample differed from Latvia and Lithuania. Parental educational level did not differ between the three samples (*χ^2^* = 10.18, *p* = 0.117). Parental relationship status differed between samples (*χ^2^* = 131.98, *p* < 0.001): more parents in Taiwan were married and fewer (compared to Latvia and Lithuania) were divorced or had never been married. Note these differences are in line with sociodemographic trends in each of the regions, e.g., Taiwan has a lower rate of out-of-wedlock births and a higher proportion of children living with two parents than two Baltic countries (Latvia and Lithuania) and an extended family form is much more prevalent [for reference see Wu et al. (40)]. There were differences between countries in the perceived family financial situation (*χ^2^* = 23.01, *p* = 0.001). Lithuanian parents reported to a greater extent that the family can afford everything and to a lower extent that the family can merely get along well enough.

**Table 1:** Demographic characteristics of the whole sample and separately for Latvia, Lithuania and Taiwan at T1.

	Total sample number (%)	Latvia number (%)	Lithuania number (%)	Taiwan number (%)
**Gender**				
Boys	333 (48.2)	110 (46.6)	101 (48.8)	122 (49.2)
Girls	358 (51.8)	126 (53.4)	106 (51.2)	126 (50.8)
**Age of the child**				
Mean	8.78	8.55	8.47	9.26
SD	0.72	0.63	0.56	0.67
**Age of the parent**				
Mean	40.00	37.48	38.71	43.53
SD	5.91	5.40	4.68	5.64
**Parental education level**				
9 years or less	13 (1.9)	4 (1.7)	3 (1.4)	6 (2.4)
10–12 years	85 (12.3)	36 (15.3)	14 (6.8)	35 (14.1)
13–15 years	116 (16.8)	39 (16.5)	41 (19.8)	36 (14.5)
16 years and more	462 (66.9)	153 (64.8)	139 (67.1)	170 (68.5)
**Parental relationship status**				
Married	535 (77.4)	137 (58.1)	171 (82.6)	227 (91.5)
Divorced, in a partnership	36 (5.2)	26 (11.0)	8 (3.9)	2 (0.8)
Never married, in a partnership	57 (8.2)	40 (16.9)	15 (7.2)	2 (0.8)
Divorced or never married, not in a partnership	48 (6.9)	26 (11.0)	6 (2.9)	16 (6.4)
Other	8 (1.2)	4 (1.7)	4 (1.9)	0 (0.0)
**Perceived family financial situation**				
We can afford all that we would like to have	26 (3.8)	3 (1.3)	15 (7.2)	8 (3.2)
We are fairly well off	378 (54.7)	122 (51.7)	113 (54.6)	143 (57.7)
We can get along well enough	254 (36.8)	103 (43.6)	72 (34.8)	79 (31.9)
Have only the very basic necessities	27 (3.9)	5 (2.1)	6 (2.9)	16 (6.5)

At Time 2 (autumn of 2019; T2), the same children and their parents were contacted to complete similar questionnaires, with a retention rate ranging from 76% in Lithuania to 85% in Taiwan and 90% in Latvia. Detailed information on the participants at T1 is provided in Table 1. There were no statistically significant differences in socio-demographics and mean scores of the main study variables (at Time 1) between those who did not complete the questionnaires at Time 2 and those who completed them.

### Instruments

The questionnaire packet (version for children and version for parents) used in the study was initially compiled in English and agreed upon during joint meetings of the research team members from all three countries/region. The research teams provided translation to the primary language in each region and/or adapted available translated versions of the questionnaire components. Several bilingual translators performed the translations, and independent back-translation was conducted to ensure the quality of the translation. The entire questionnaire packet was pilot tested in each participant country/region (with samples of *N* = 50 children and *N* = 50 parents in each of the three countries) and refined if needed before the actual study began.

The *Compulsive Internet Use Scale* (CIUS) with 14 items was used to assess the child’s problematic internet use (14, 30). The child and the child’s parent completed nearly identical versions of the CIUS, with modifications from first to third person (e.g., *“Do you find it difficult to …?” – “Does your child find it difficult to …?”*). Both the child and parent (on separate forms) rated the items on a 5-point scale ranging from 0 (*never*) to 4 (*very often*). For the Taiwanese sample, we used the Chinese version translated and validated by Dhir et al. (13) with Taiwanese high school students. In Latvia and Lithuania, the CIUS was independently translated by several bilingual translators to Latvian and Lithuanian for the purposes of this study. A back translation was also performed. A consensus agreement was reached in case of any discrepancies. The translated versions were tested in the pilot study and revealed good psychometric characteristics for parental and child reports. The final psychometric characteristics of this scale for parental and child reports are presented in the results.

The *Strengths and Difficulties Questionnaire* [SDQ (41)] with 25 items was completed by the parent to assess the child’s emotional and behavioral problems, as well as prosocial behavior. Parents rated the items on a 3-point scale ranging from 0 (*not true*) to 2 (*certainly true*). In this study we used data from 20 items comprising four subscales (conduct problems, emotional symptoms, problems with peers, hyperactivity/inattention). These items combined allowed for the calculation of the total problems score. We used the Latvian, Lithuanian, and Chinese validated versions of the SDQ that were already available (49–51). At Time 1, Cronbach’s alpha for the total SDQ scale was 0.68 and the McDonald Omega was 0.69. For subscales Cronbach’s alpha was from 0.52 to 0.69 and the McDonald Omega was from 0.53 to 0.69. At Time 2, Cronbach’s alpha for the total SDQ scale was 0.69 and the McDonald Omega was 0.69. For subscales Cronbach’s alpha was from 0.59 to 0.70 and the McDonald Omega was from 0.61 to 0.73.

*Time online* was assessed with the child and parents (on separate forms) providing answers to the following two questions: “About how long do you (does your child) spend on the internet per day during a regular weekday (school day)?” and “About how long do you (does your child) spend on the internet per day during a regular weekend day or holiday?” The choices for answers for each question were presented on a 9-point scale: 1 (little or no time), 2 (half an hour), 3 (1 h), 4 (2 h), 5 (3 h), 6 (4 h), 7 (5 h) 8 (6 h), and 9 (7 h or more).

### Procedure

The study procedure was conducted as follows: (1) permission was received from the school principals to conduct the research in their school; (2) written invitations to participate in the study were sent out to the parents/primary caregivers of all children in the designated classrooms; (3) the parents who agreed to participate signed the informed consent, completed the parent questionnaire, and the child returned the completed questionnaire to the school in an enclosed envelope; (4) the children, whose parents have provided written consent, and whom themselves agreed to take part in the study, completed the child self-report questionnaire during a classroom period at school [for more information on procedure (22, 40)]. The percentage of parents who responded to the initial invitation with written consent for their child and themselves to participate in the study at Time 1 was 65% in Lithuania and 69% in Latvia and Taiwan.

This study received ethics committee approval from the University of Latvia in Latvia (07.11.18. V69/15), Vilnius University in Lithuania (2018-10-12, no. 18), and National Taiwan University in Taiwan (201705ES030).

### Data analyses

We conducted a confirmatory factor analysis (CFA) and cross-lagged analysis using Mplus 8.2. to examine the psychometric properties of the CIUS and the relationships between parent-report and child self-report CIUS over time. We used FIML (Full Information Maximum Likelihood) estimator and the raw data (some data were missing) as the input file for all the models in the Mplus software. Missingness occurred due to not having answers to some questions and thus were treated as missing at random. The proportion of missing values was calculated using a covariance “coverage” matrix in Mplus, which estimates available observations for each pair of variables. The minimum coverage for the analyses is 0.10. In this study the coverage in the tested models ranged from 0.90 to 1.00. All the models tested in this study were evaluated using several goodness-of-fit indices: the Chi-square (χ^2^) test and its value of p, CFI (Comparative Fit Index); TLI (Tucker-Lewis Index); RMSEA (Root Mean Square Error of Approximation) and its value of p; and SRMR (Standardized Root Mean Residual). CFI and TLI values greater than 0.90 represent an adequate fit to the data (52) and values greater than 0.95 suggest a very good model fit (53). RMSEA and SRMR values less than 0.08 represent a reasonable model, and values less than 0.05 indicate a close model fit with the data (54). Considering that there is no one good fit index that would indicate a good model fit, for the evaluation of final models, we used all the fit indices mentioned above. We also used a chi-square difference test between two nested (competing factor structures) models. We calculated measurement invariance for the CIUS scales in all three languages.

Data was also analyzed using the statistical package SPSS. For mean value comparisons between Time 1 and Time 2 ratings, we used paired sample t-test, and for associations between variables – Pearson correlation. Two scales from SDQ (conduct problems and hyperactivity/inattention at T1 and T2) were non-normally distributed and were transformed (by using logarithmic transformation) for comparing mean values and calculation correlations.

## Results

### Child self-report data

We followed the primary factor structure specified by the CIUS authors, including the 14 observed variables and one latent factor. The initial model for the whole sample of children at T1 revealed that the model had an adequate fit. In Table 2, fit indices for all the models are presented. However, four items had very low factor loadings (from 0.10 to 0.30; see factor loadings for this model presented in Table 3). Thus, items 3, 4, 8, and 9 were removed from the model, and further analysis on the child-reported CIUS data were conducted upon the premise of testing the model fit of this proposed 10-item CIUS scale. To improve the model fit, several correlations between error variances were added: e1–e2; e6–e7; e6–e11; e7–e14; e1–e13. To correlate endogenous (observed) variables in the model, we conducted an overview of the error terms. This we based upon the premise that if some items are highly correlated in the model, they often share similar errors. Adding correlations between error variances in the model would not change results, it just would improve the model fit. The modified model showed a good fit. All the nested models (competing factor structures models) at T1 were compared by chi-square difference tests. All the models differed significantly (Table 2). Model fit information for separated Lithuanian, Latvian, and Taiwanese samples is presented in Table 2. The factor loadings for the whole sample and each data collection site are presented in Table 4. Cronbach’s alpha for 10 CIUS items of the children’s data at T1 was high (0.87), and the McDonald Omega was 0.88. The T2 model for all 14 child-reported items of the CIUS for the whole sample initially had a poor model fit (Table 2). Again, several items 3, 8, and 9 had very low factor loadings (from 0.19 to 0.32) and were removed. Although item 4 had a factor loading of 0.53, it was also removed in order to have the same items at T1 and T2. With four items removed, the model fit remained poor (Table 2). To improve the model, several correlations between error variances were added: e1–e2; e6–e7; e7–e10; e12–e13. The modified model had a good fit. All of the nested models (competing factor structures models) at T2 were compared by chi-square difference tests. All of the models differed significantly (Table 2). Model fit information for separated Lithuanian, Latvian, and Taiwanese samples in T2 is presented in Table 2. The factor loadings are presented in Table 4. The internal consistency for the 10 items at T2 was high (0.87), and the McDonald Omega was 0.90.

**Table 2:** Fit indices for tested models of the child self-report and parent-report CIUS scores.

Model	χ^2^ (df)	Δχ^2^ (Δdf)	*p*	CFI	TLI	RMSEA	SRMR
**Child self-report CIUS models**							
One factor with all items without correlated errors T1	405.32 (77)	–	<0.001	0.90	0.88	0.07	0.05
One factor without items 3, 4, 8, 9 and without correlated errors T1	211.26 (35)	194.06 (42)	<0.001	0.94	0.92	0.08	0.04
One factor without items 3, 4, 8, 9 and with correlated errors T1	58.35 (30)	152.91 (5)	0.002	0.99	0.99	0.03	0.02
One factor without items 3, 4, 8, 9 and with correlated errors T1 (only Latvian sample)	45.07 (30)	–	0.038	0.98	0.97	0.05	0.03
One factor without items 3, 4, 8, 9 and with correlated errors T1 (only Lithuanian sample)	52.58 (30)	–	0.006	0.97	0.95	0.06	0.04
One factor without items 3, 4, 8, 9 and with correlated errors T1 (only Taiwanese sample)	28.79 (30)	–	0.529	1.00	1.00	0.00	0.03
One factor with all items without correlated errors T2	652.43 (77)	–	<0.001	0.83	0.80	0.10	0.06
One factor without items 3, 4, 8, 9 and without correlated errors T2	435.30 (35)	217.13 (42)	<0.001	0.86	0.82	0.12	0.06
One factor without items 3, 4, 8, 9 and with correlated errors T2	68.35 (31)	366.95 (4)	<0.001	0.99	0.98	0.04	0.02
One factor without items 3, 4, 8, 9 and with correlated errors T2 (only Latvian sample)	59.81 (31)	–	0.001	0.97	0.95	0.06	0.04
One factor without items 3, 4, 8, 9 and with correlated errors T2 (only Lithuanian sample)	58.41 (31)	–	0.002	0.96	0.94	0.07	0.05
One factor without items 3, 4, 8, 9 and with correlated errors T2 (only Taiwanese sample)	96.99 (31)	–	<0.001	0.94	0.92	0.09	0.04
**Parent-report CIUS models**							
One factor without correlated errors T1	1151.28 (77)	–	<0.001	0.83	0.79	0.13	0.06
One factor with correlated errors T1	261.44 (67)	889.84 (10)	<0.001	0.97	0.96	0.06	0.03
One factor with correlated errors T1 (only Latvian sample)	91.11 (67)	–	0.027	0.98	0.98	0.04	0.04
One factor with correlated errors T1 (only Lithuanian sample)	163.82 (67)	–	0.001	0.94	0.92	0.08	0.05
One factor with correlated errors T1 (only Taiwanese sample)	157.98 (67)	–	<0.001	0.95	0.93	0.07	0.04
One factor without correlated errors T2	1076.25 (77)	–	<0.001	0.82	0.79	0.14	0.06
One factor with correlated errors T2	202.52 (68)	873,73 (9)	<0.001	0.98	0.97	0.05	0.03
One factor with correlated errors T2 (only Latvian sample)	122.14 (68)	–	<0.001	0.97	0.95	0.06	0.04
One factor with correlated errors T2 (only Lithuanian sample)	185.53 (68)	–	<0.001	0.94	0.92	0.09	0.05
One factor with correlated errors T2 (only Taiwanese sample)	160.51 (68)	–	<0.001	0.95	0.94	0.07	0.04

**Table 3:** Factor loadings of 14 CIUS items (child self-report data) for the whole sample and for Latvia, Lithuania, and Taiwan separately at T1/T2, respectively.

CIUS items	Total sample	Latvia	Lithuania	Taiwan
1 “Do you find it difficult to stop using the Internet when you are online?”	0.62/0.59	0.56/0.55	0.63/0.61	0.66/0.65
2 “Do you continue to use the Internet despite your intention to stop?”	0.60/0.59	0.55/0.58	0.61/0.59	0.68/0.64
3 “Do others (e.g., parents, teachers, others) say you should use the Internet less?”	0.29/0.36	0.29/0.42	0.31/0.43	0.26/0.31
4 “Do you prefer to use the Internet instead of spending time with others (e.g., friends, parents)?”	0.39/0.53	0.34/0.49	0.40/0.53	0.45/0.54
5 “Are you short of sleep because of the Internet?	0.49/0.55	0.40/0.47	0.42/0.47	0.58/0.63
6 “Do you think about the Internet, even when not online?”	0.66/0.69	0.62/0.71	0.64/0.67	0.68/0.67
7 “Do you look forward to your next Internet session?”	0.70/0.71	0.70/0.72	0.71/0.72	0.70/0.65
8 “Do you think you should use the Internet less often?”	0.10/0.19	0.10/0.23	0.17/0.23	0.10/0.18
9 “Have you unsuccessfully tried to spend less time on the Internet?”	0.44/0.34	0.38/0.34	0.53/0.44	0.48/0.46
10 “Do you rush through your homework in order to go on the Internet?”	0.66/0.66	0.64/0.67	0.62/0.57	0.70/0.70
11 “Do you neglect your daily obligations (at school or at home) because you prefer to go on the Internet?”	0.61/0.61	0.53/0.59	0.60/0.58	0.65/0.63
12 “Do you go on the Internet when you are feeling down?”	0.63/0.61	0.52/0.58	0.66/0.62	0.70/0.62
13 “Do you use the Internet to escape from your sorrows or get relief from negative feelings?”	0.64/0.66	0.57/0.64	0.68/0.62	0.67/0.72
14 “Do you feel restless, frustrated, or irritated when you cannot use the Internet?”	0.69/0.69	0.68/0.69	0.68/0.63	0.67/0.74

**Table 4:** Factor loadings of 10 retained CIUS items (child self-report data) for the whole sample and for Latvia, Lithuania, and Taiwan separately at T1/T2, respectively.

CIUS items	Total sample	Latvia	Lithuania	Taiwan
1 “Do you find it difficult to stop using the Internet when you are online?”	0.59/0.56	0.55/0.53	0.61/0.56	0.60/0.58
2 “Do you continue to use the Internet despite your intention to stop?”	0.58/0.57	0.54/0.56	0.60/0.55	0.64/0.59
5 “Are you short of sleep because of the Internet?	0.48/0.56	0.42/0.49	0.43/0.47	0.58/0.63
6 “Do you think about the Internet, even when not online?”	0.67/0.66	0.64/0.71	0.66/0.63	0.68/0.63
7 “Do you look forward to your next Internet session?”	0.67/0.67	0.68/0.71	0.69/0.65	0.61/0.60
10 “Do you rush through your homework in order to go on the Internet?”	0.68/0.67	0.67/0.70	0.65/0.58	0.70/0.70
11 “Do you neglect your daily obligations (at school or at home) because you prefer to go on the Internet?”	0.64/0.64	0.58/0.64	0.61/0.62	0.68/0.65
12 “Do you go on the Internet when you are feeling down?”	0.60/0.54	0.52/0.54	0.64/0.55	0.66/0.56
13 “Do you use the Internet to escape from your sorrows or get relief from negative feelings?”	0.62/0.61	0.56/0.62	0.66/0.57	0.64/0.67
14 “Do you feel restless, frustrated, or irritated when you cannot use the Internet?”	0.68/0.73	0.69/0.73	0.69/0.65	0.64/0.77

Excluded are the following items: item 3. “Do others (e.g., parents, teachers, others) say you should use the Internet less?”; item 4. “Do you prefer to use the Internet instead of spending time with others (e.g., friends, parents)?”; item 8. “Do you think you should use the Internet less often?”; and item 9. “Have you unsuccessfully tried to spend less time on the Internet?”

To test construct stability in time (over a one-year period), the autoregressive model was examined: the child-reported CIUS at T1 (with 10 items with correlated error variances for T1) predicted child-reported CIUS at T2 (with 10 items with correlated error variances for T2) for the whole sample. The model showed an adequate fit (χ^2^_160_ = 440.81, *p* < 0.001; CFI = 0.95; TLI = 0.95; RMSEA = 0.05). Correlations between error variances e5-e7 over time were included and this final model showed a rather good fit (χ^2^_158_ = 372.84, p < 0.001; CFI = 0.96; TLI = 0.96; RMSEA = 0.04). Stability between CIUS at T1 and T2 was high (Est = 0.61***). The chi-square difference test (Δχ^2^ = 11.56, Δdf = 2) of the multigroup model between different countries/regions showed one difference between stability on the paths; for the Taiwanese children, the stability of the CIUS was much higher over time. It seems that the CIUS is rather stable over a 1-year period for child self-report.

### Parent-report data

A CFA for all 14 parent-reported items of the CIUS for the whole sample at T1 revealed that the model had a poor fit (Table 2). Several modifications were suggested between item error variances: e1–e2; e1–e7; e3–e8; e5–e13; e6–e7; e7–e10; e7–e14; e9–e10; e10–e11; e12–e13. Entering the correlated error variances resulted in a good model fit. Both nested models (competing factor structures models) at T1 were compared by chi-square difference tests. All of the models differed significantly (Table 2). Model fit information for separated Lithuanian, Latvian, and Taiwanese samples is presented in Table 2. The factor loadings for the whole sample and each country/region are presented in Table 5. Cronbach’s alpha for the whole sample parents’ data at T1 was high (0.92), and the McDonald Omega was 0.92. The T2 model for parent-report data without any modifications showed a poor fit (Table 2). There were several modifications suggested between error variances: e1–e2; e1–e3; e1–e7; e2–e3; e2–e8; e3–e8; e6–e7; e10–e11; e12–e13. The modified model indicated a good fit. The factor loadings are in Table 5. Model fit information for separated Lithuanian, Latvian, and Taiwanese samples at T2 is presented in Table 2. The internal consistency for all 14 items at T2 was high (0.92), and the McDonald Omega was 0.91.

**Table 5:** Factor loadings of 14 CIUS items (parent-report data) for the whole sample and for Latvia, Lithuania, and Taiwan separately at T1/T2, respectively.

CIUS items *How often does your child …*	Total sample	Latvia	Lithuania	Taiwan
1 “… find it difficult to stop using the Internet when she/he is online”	0.63/0.67	0.57/0.65	0.61/0.71	0.57/0.65
2 “… continue to use the Internet despite your request to stop”	0.69/0.62	0.64/0.64	0.65/0.63	0.67/0.60
3 “… do you or others (e.g., your partner) say that your child should use the Internet less”	0.64/0.61	0.63/0.62	0.65/0.66	0.54/0.54
4 “… prefer to use the Internet instead of spending time with others (e.g., siblings, parents, friends)”	0.75/0.76	0.73/0.72	0.73/0.76	0.73/0.80
5 “… is short of sleep because of the Internet”	0.54/0.60	0.40/0.54	0.50/0.58	0.58/0.62
6 “…think about the Internet, even when not online”	0.69/0.71	0.59/0.69	0.69/0.67	0.70/0.73
7 “… look forward to her/his next Internet session”	0.68/0.74	0.64/0.76	0.70/0.75	0.60/0.69
8 “… do you think the child should use the Internet less often”	0.61/0.56	0.73/0.66	0.74/0.75	0.52/0.52
9 “… has your child has unsuccessfully tried to spend less time on the Internet”	0.61/0.60	0.49/0.55	0.67/0.62	0.61/0.60
10 “… rush through her/his home work in order to go on the Internet”	0.70/0.73	0.61/0.70	0.68/0.70	0.67/0.72
11 “… neglect her/his daily obligations (work, school, or family life) because she/he prefers to go on the Internet”	0.69/0.70	0.56/0.64	0.70/0.71	0.70/0.76
12 “… go on the Internet when she/he is feeling down”	0.64/0.65	0.51/0.61	0.62/0.65	0.69/0.66
13 “…use the Internet to escape from her/his sorrows or get relief from negative feelings”	0.63/0.64	0.49/0.56	0.62/0.63	0.69/0.67
14 “… feel restless, frustrated, or irritated when he/she cannot use the Internet”	0.76/0.80	0.70/0.77	0.76/0.81	0.72/0.79

In this study, we tested factorial invariance between different countries/sites, and thus configural, metric, scale invariance for child self-report and parent-report data are presented in Table 6. Results in Table 6 suggest that configural invariance was supported. However, differences between configural and metric models supported metric invariance. ΔCFA and ΔRMSEA between the metric and scalar models indicate a lack of scalar invariance. Thus, results suggest similar items measuring CIUS constructs by children and their parents, but factor loadings of those items were not equivalent across the three languages. This is understandable, considering that children from very different countries were evaluated. Results also suggest that participants who had the same value on the latent CIUS construct did not have equal values on the items from which the construct is based.

**Table 6:** Measurement invariance procedure conducted between CIUS in different languages.

Model	χ^2^ (df)	Δχ^2^ (Δdf)	RMSEA	p	CFI	TLI	SRMR
**Children’s model T1**							
Configural invariance	133.98 (90)	–	0.04	0.825	0.98	0.98	0.03
Metric invariance	185.43 (108)	51.46 (18)	0.05	0.464	0.97	0.97	0.06
Scalar invariance	278.65 (126)	93.22 (18)	0.07	0.008	0.95	0.94	0.07
**Children’s model T2**							
Configural invariance	228.20 (93)	–	0.08	0.001	0.95	0.93	0.04
Metric invariance	282.91 (111)	54.71 (18)	0.08	<0.001	0.94	0.93	0.07
Scalar invariance	428.91 (129)	146.01 (18)	0.10	<0.001	0.90	0.89	0.07
**Parents model T1**							
Configural invariance	455.53 (201)	–	0.07	<0.001	0.96	0.94	0.04
Metric invariance	614.71 (227)	159.18 (26)	0.08	<0.001	0.94	0.92	0.09
Scalar invariance	614.71 (227)	159.18 (26)	0.07	<0.001	0.94	0.92	0.09
**Parents model T2**							
Configural invariance	484.20 (204)	–	0.08	<0.001	0.95	0.94	0.04
Metric invariance	641.63 (230)	157.43 (26)	0.09	<0.001	0.93	0.92	0.10
Scalar invariance	1010.19 (256)	368.56 (26)	0.11	<0.001	0.87	0.86	0.13

In order to evaluate the stability of parent-reported CIUS over time, we ran an autoregressive model with parent-reported CIUS at Time 1 (14 items with correlated error variances for T1), predicting T2 latent CIUS (14 items with correlated error variances for T2) for the whole sample. The autoregressive model showed an adequate fit (χ^2^_330_ = 1,494.69, *p* < 0.001; CFI = 0.91; TLI = 0.90; RMSEA = 0.06); however, some correlations between error variances were suggested for items 1, 3, 4, 5, 7, 8, 9, 10, and 11 over time; and also, between e3 of T1 and e8 of T2, and e8 of T1 and e3 of T2. The final model with correlated errors over time showed a good fit (χ^2^_330_ = 939.46, *p* < 0.001; CFI = 0.95; TLI = 0.94; RMSEA = 0.05). The stability between T1 and T2 of the CIUS was high (Est = 0.70***). The multigroup comparison model between different countries/regions suggests that stability paths are similar. The chi-square difference test showed two differences between factor loadings of items 1 and 2 over time (Δχ^2^ = 4.06, Δdf = 1; Δχ^2^ = 4.05, Δdf = 1, for items 1 and 2, respectively) and also differences between countries for factor loadings of items 1 and 2 over time (Δχ^2^ = 39.53, Δdf = 2 for item 1; Δχ^2^ = 39.50, Δdf = 2 for item 2). It seems that some items of the CIUS were not very stable over the one-year period for the parent reports.

### Parent-report and child self-report data

Descriptive statistics for the final CIUS scales (for children 10 items and for parents 14 items), and for all other measures used in the analyses are presented in Table 7. We did not find any differences in comparing the mean values of CIUS over time for child self-report data or parent-report data. Child self-reported duration of time online increased significantly over time (*t* = −2.45, *p* = 0.014 weekdays; *t* = −3.42, *p* = 0.001 weekends). Parent-reported child’s duration of time online over time increased as well (*t* = −6.43, *p* < 0.001 weekdays; *t* = −4.67, *p* < 0.001 weekends). Of the SDQ ratings, we found that only child hyperactivity scores differed over time: at T2, parents evaluated hyperactivity lower at T1 (*t* = 2.59, *p* = 0.010). Comparing the mean values of child self-report and parent-reported CIUS ratings at T1, we did find significant differences (*t* = 6.43, *p* < 0.001), indicating that parents reported their child to have higher CIUS scores than their child’s self-reported CIUS scores at T1. At T2, we see the same significant difference between child self-report and parent-reported CIUS scores (*t* = 8.51, *p* < 0.001), suggesting the same tendency. Comparing child self-reported and parent-reported duration of time online at T1, there were no significant differences in time reported during weekdays (*t* = −1.68, *p* = 0.093). Still, there was a significant difference in time reported during weekends (*t* = −2.64, *p* = 0.008), indicating that parents consider that their children spend longer time online during weekends than do the children themselves. Child self-reported and parent-reported duration of time online at T2 comparison showed significant differences in time online reported during weekdays (*t* = −3.82, *p* < 0.001) and weekends (*t* = −2.48, *p* = 0.013), indicating that parents consider that their children spend longer time online during weekdays and weekends than do the children themselves.

**Table 7:** Descriptive statistics for all of the study variables at T1 and T2.

	T1				T2			
	Min	Max	M	SD	Min	Max	M	SD
**CIUS**								
CIUS child report	0	3.90	1.01	0.85	0	3.60	0.98	0.77
CIUS parent report	0	3.64	1.25	0.76	0	3.50	1.26	0.74
**Time online (parent report)**								
Weekdays	1	7	2.50	1.37	1	9	2.82	1.52
Weekends	1	9	3.76	1.84	1	9	4.07	1.84
**Time online (child report)**								
Weekdays	1	9	2.37	1.76	1	9	2.57	1.77
Weekends	1	9	3.52	2.28	1	9	3.88	2.22
**SDQ (parent report)**								
Emotional symptoms	0	9	2.11	1.84	0	9	2.10	1.85
Conduct problems	0	10	2.43	1.34	0	8	2.34	1.19
Hyperactivity/inattention	0	10	4.24	1.55	0	9	4.07	1.58
Peer relationship problems	1	10	4.52	1.32	0	8	4.50	1.89
Total	6	39	13.31	4.11	2	29	13.01	4.04

Min, minimum value of the variable; Max, maximum value of the variable; M, mean value; SD, standard deviation.

Correlational analysis showed that the CIUS scores, reported by parents and children were associated at T1 (*r* = 0.39***) and T2 (*r* = 0.41***). These correlations indicate that the parents and their child did not answer identically, therefore, information provided by the child cannot simply replace the parent-reported data, nor vice versa. We also used a cross-lagged model to examine if parent-reported CIUS could predict child-self-reported CIUS over time and vice versa. The model that included the 14 parent-reported CIUS items and the 10 children self-reported CIUS items at T1 and T2 (with all the error variance correlations from the autoregressive models) showed an acceptable fit (χ^2^_1,033_ = 2,026.23 *p* < 0.001; CFI = 0.94; TLI = 0.94; RMSEA = 0.04). Analysis of data from the whole sample (see Table 8) suggests that parent-reported CIUS can predict child self-reported CIUS over time and child self-reported CIUS can predict parent-reported CIUS. The Chi-square difference test (Δχ^2^ = 6.24, Δdf = 2) of the multigroup model between different countries/regions suggests that Taiwanese children self-reported CIUS could predict parent-reported CIUS over time, while in Latvia and Lithuania, these paths were not significant. At T2, the association between child-self-reported and parent-reported CIUS was significantly lower than at T1 for the whole sample (Δχ^2^ = 36.97, Δdf = 1), but it was not significantly different by country/region. Results suggest that the similarities between parent-report and child self-report might become less with the child’s increasing age because the association between parent-reported and child-self-reported CIUS ratings is much lower at T2 (when the child is older) compared to T1.

**Table 8:** Standardized estimates of stability and cross-paths for the cross-lagged panel model for the whole sample and for each country/region separately.

	Total sample	Latvia	Lithuania	Taiwan
**Parent stability path**				
CIUS T1–CIUS T2	0.65^***^	0.64^***^	0.63^***^	0.60^***^
**Child’s stability path**				
CIUS T1–CIUS T2	0.55^***^	0.49^***^	0.51^***^	0.66^***^
**Parent to child cross path**				
Parent CIUS T1–child CIUS T2	0.15^***^	0.11	0.16^*^	0.16^*^
**Child to parent cross path**				
Child CIUS T1–parent CIUS T2	0.12^**^	0.03	0.10	0.23^***^
**Correlations at one time point**				
Child CIUS T1–parent CIUS T1	0.44^***^	0.39^***^	0.33^***^	0.46^***^
Child CIUS T2–parent CIUS T2	0.20^***^	0.39^***^	0.27^**^	0.20^*^

^*^p < 0.05; ^**^p < 0.01; ^***^p < 0.001.

To further validate the child self-reported and parent-reported CIUS versions, we ran correlations between the CIUS mean scores at each time point and SDQ and time online ratings at T1 and T2. The results (Table 9) show correlations between parent-reported CIUS and SDQ subscales and between parent-reported CIUS and parent-reported child’s time online. Child-reported CIUS was also associated with parent-reported SDQ subscales and parent-reported time online.

**Table 9:** Correlations between CIUS (parent and child reported), SDQ subscales, and time online.

	Parent-reported CIUS (14 items) T1	Parent-reported CIUS (14 items) T2	Child-reported CIUS (10 items) T1	Child-reported CIUS (10 items) T2
**SDQ**				
Emotional symptoms	0.31^***^	0.29^***^	0.13^**^	0.14^***^
Conduct problems	0.32^***^	0.31^***^	0.21^***^	0.19^***^
Hyperactivity/inattention	0.30^***^	0.32^***^	0.18^***^	0.19^***^
Peer relationship problems	0.16^***^	0.13^**^	0.05	0.02
Total problems	0.41^***^	0.40^***^	0.22^***^	0.21^***^
**Time online (parent report)**				
Weekdays	0.48^***^	0.44^***^	0.29^***^	0.29^***^
Weekends	0.47^***^	0.46^***^	0.28^***^	0.30^***^
**Time online (child report)**				
Weekdays	0.24^***^	0.25^***^	0.40^***^	0.32^***^
Weekends	0.25^***^	0.26^***^	0.46^***^	0.47^***^

^**^p < 0.01; ^***^p < 0.001.

## Discussion

The internet has become an integral part of children’s lives worldwide, but there is still a lack of sufficient data on how children use the internet and with what consequences. Despite a plentitude of studies reporting the pros and cons of excessive internet use by adolescents, the internet use of primary school-aged children is under-researched, mainly due to methodological concerns and doubts on whether children’s self-reports can be used when researching their internet use. To our knowledge, this study is the first to date that aimed to analyze the psychometric characteristics of the Compulsive Internet Use Scale ratings as reported by 8-to-10-year-old children themselves.

The child self-reported CIUS showed good psychometric properties with the 10-item version when items 3, 4, 8, and 9 of the original 14-item scale were excluded. Importantly, the remaining ten items reflect all five of the major symptoms of the internet use compulsivity as initially proposed by Meerkerk et al. (14, 30). Moreover, the same items had been excluded by other researchers who were developing short forms to be used with adolescents and/or adults. The factor loading for CIUS item 8 (*Do you think you should use the Internet less often?*) was similarly low in a study that used the English version of the CIUS in a sample of Indian adolescents aged 12–19 years old, indicating that also in other contexts this item has a small effect on the total CIUS score (33). In congruence with other studies, items 4, 8, and 9 were not included in the CIUS-7 and CIUS-5 (26). Similarly, items 3 and 8 were not retained in the short form developed by Gmel et al. (12) It seems that items involving the thought or behaviors of others (e.g., items 3 and 4) or self-consciousness (e.g., items 8 and 9) can be more challenging for study participants or leave greater room for interpretation, especially in studies such as ours with young school-aged children.

In our study the construct validity of the child’s self-reported CIUS was supported by correlations with time online as provided by the children and their parents and with the child’s behavioral and emotional problems, as rated by their parents. The child self-reported CIUS ratings were relatively stable over the 1 year period for children in each of the three countries/regions—Latvia, Lithuania, and Taiwan, with the questionnaires completed in three different languages—Latvian, Lithuanian, and Chinese. The scale reliability at all measurement points remained very good. Thus, based on the results of our study, the child self-report CIUS was shown to be a reliable and sufficiently valid instrument for the self-assessment of problematic internet use in three different cultural samples, therefore adding to similar evidence from other studies with adolescents and young adults from the past several years (26, 34, 38). In addition, the results of our study supported stability of the child-reported CIUS across time, and therefore added to the considerable lack of test–retest measurements or longitudinal studies in evaluating the consistency and continuity of the PUI over time (4, 9).

The results of measurement invariance across the three languages in our study revealed good metric invariance, despite the unsatisfactory scalar invariance of the child-reported 10-item CIUS and parent-reported 14-item CIUS. Similarly, in previous study (26) with adult samples poor invariance for the full CIUS-14, although sufficient metric (but not scalar) invariance for the short forms of the CIUS were shown when tested in several languages. Gmel et al. (12) confirmed the weak (metric) and strong (scalar) invariance for shorter form of CIUS reported in several languages from one cultural sample. Fineberg et al. in their complete review of the PUI have also stressed the insufficient efforts to confirm or failure to establish the measurement invariance across different countries and cultures (4). Thus, further studies should elaborate on the most applicable culture/language and child’s age invariant forms for children and for parents/caregivers reporting the child’s problematic internet use.

We also provided support for the reliability and validity of the parent-reported 14-item CIUS that assesses parental perceptions of their child’s internet use compulsivity. Importantly, our study also showed that parent-reported CIUS ratings could predict child-reported CIUS over time and child self-reported CIUS could predict parent-reported CIUS, indicating the appropriate specificity of the instrument. At the same time, the results of our study also indicate that the parent and child-reported information may reflect different perspectives, and therefore implies that parent-reported information cannot be simply replaced by child-reported information about their internet use compulsivity, and vice versa. Results further suggest that similarities between parent and child reports might decrease with an increase in the child’s age.

Our findings support similar affirmations from other studies that parents can provide comparable, yet different, information than their child (47, 48) and that agreement between parental and child reports often is less pronounced when reporting mental processes rather than observable behaviors (44, 49) or when reporting context-specific (e.g., home vs. school) behaviors and concerns (55). The multi-informant approach of gathering information from various sources on children’s and adolescents’ behavior and health issues is highly recommended in clinical practice. Thus, further studies providing a greater understanding of cross-informant integration (56) and management of informant discrepancies (55) are needed in developmental psychopathology, including that which relates to internet-use addictions. Keeping in mind the addictive features of online behaviors (4, 10, 15), our results also suggest possible advantages of using reports by proxy (e.g., reports by close family members or friends) in combination with self-reports when researching or screening for adults’ problematic online behaviors as well.

Our study’s non-representative and medium sample size poses limitations for generalizing our results. Also, our non-representative sample does not allow us to determine a valid screening cutoff point for identifying those primary school-aged children who are at especially high risk for problematic internet use. Replications in other cultures and with larger samples, as well as the use of additional procedures to test for construct validity (such as factorial invariance) and content validity, are highly encouraged. Another limitation of this study is that we used self-report and parental reports for the duration of time spent online (instead of the objectively measured). In addition, some subscales of the SDQ used to assess behavioral and emotional problems had satisfactory reliability. Thus, further validation of the CIUS should also include testing the associations with more reliable measures for the time spent online and for mental health problems or problem behaviors. Future studies should also strive to identify a cutoff point for clinically problematic CIUS scores in different cultures and with different age groups (33, 34), e.g., non-referred and referred children and adolescents, as well as to continue investigation of the predictive power of the PUI, using the CIUS, on significant outcome variables (26).

This study’s strength is that it provides evidence that children as young as 8–10 years old can reliably and consistently provide valuable information on their compulsive tendencies to use the internet. Furthermore, our study confirms the associations between compulsive internet use and emotional and behavioral problems in primary school-aged children. This accentuates the importance of further research aiming to reveal the antecedents and consequences of problematic internet use by children. There is a high need for the early detection and recognition of PUI (4), as well as a need for more thorough identification of early predictors of PUI, especially with the consideration of contextual factors such as family (9). Thus, we encourage further studies to benefit from the possibility of including both child-report and parent-report, thereby providing multiple and more complete perspectives in furthering our understanding of PUI, with the overarching aim of providing greater possibilities for prevention and treatment.

## Data availability statement

The raw data supporting the conclusions of this article will be made available by the authors, without undue reservation.

## Ethics statement

The studies involving human participants were reviewed and approved by Ethics committee from the University of Latvia in Latvia (07.11.18. V69/15), Vilnius University in Lithuania (2018-10-12, no. 18), and National Taiwan University in Taiwan (201705ES030). Written informed consent to participate in this study was provided by the participants’ legal guardian/next of kin.

## Author contributions

All authors listed have made a substantial, direct, and intellectual contribution to the work and approved it for publication.

## Funding

This study and the project were supported by Mutual Funds between the Ministry of Education and Science of the Republic of Lithuania, the Ministry of Education and Science of the Republic of Latvia, and the Ministry of Science and Technology (MOST) of the Republic of China (Taiwan). In Lithuania, research was funded by MOST and by Research Council of Lithuania (contract no. S-LLT-18-3). In Latvia, research was funded by MOST and the Latvia Ministry of Education and Science (VIAA contract no. LV-LT-TW/2018/2). In Taiwan, research was funded by the Ministry of Science and Technology, Taiwan (MOST 107-2923-H-152-001-MY3).

## Conflict of interest

The authors declare that the research was conducted in the absence of any commercial or financial relationships that could be construed as a potential conflict of interest.

## Publisher’s note

All claims expressed in this article are solely those of the authors and do not necessarily represent those of their affiliated organizations, or those of the publisher, the editors and the reviewers. Any product that may be evaluated in this article, or claim that may be made by its manufacturer, is not guaranteed or endorsed by the publisher.
